# Detailed Analysis of the Debris-Fretting Damage Areas on Coated Fuel Cladding

**DOI:** 10.3390/ma18010143

**Published:** 2025-01-02

**Authors:** Ondřej Pašta, Marcin Kopeć, Ladislav Cvrček, Jakub Krejčí, Patricie Halodová, Kristína Sihelská

**Affiliations:** 1 Centrum Výzkumu Řež s.r.o., Hlavní 130, 250 68 Husinec-Řež, Czech Republic; marcin.kopec@cvrez.cz (M.K.);; 2 Department of Materials Engineering, Faculty of Mechanical Engineering, Czech Technical University in Prague, Karlovo Náměstí 293/13, Prague 2, 120 00 Prague, Czech Republic; 3 UJP PRAHA, a.s., Praha-Zbraslav, 156 10 Prague, Czech Republic

**Keywords:** cladding, coating, debris fretting, nuclear fuel damage

## Abstract

Fuel failure caused by fretting damage to cladding remains a relevant issue despite decades of research and development aimed at enhancing the physical parameters of fuel. This paper presents the results of experiments conducted at the Research Centre Řež on Zr-1%Nb alloy tube specimens covered with protective coatings made of chromium (Cr) and nitrogen (N) compounds. The experiments involved debris-fretting tests under dry conditions at room temperature as well as microscopic measurements of groove depths. A detailed analysis was performed using the Scanning Electron Microscopy, Energy-Dispersive X-Ray Spectroscopy, Electron Backscatter Diffraction, and Focused Ion Beam techniques. The objectives of the tests were (1) to compare the debris-fretting resistance between the reference Zr-1%Nb specimens and those of the same alloy coated with various compositions, and (2) to demonstrate the positive effects of coating applications on the endurance of fuel cladding. The conducted analysis revealed a significant advantage in using cladding with a thin, wear-resistant layer compared to standard cladding material, with the CrN-coated specimens exhibiting 36 times better fretting resistance.

## 1. Introduction

**Figure 1:**
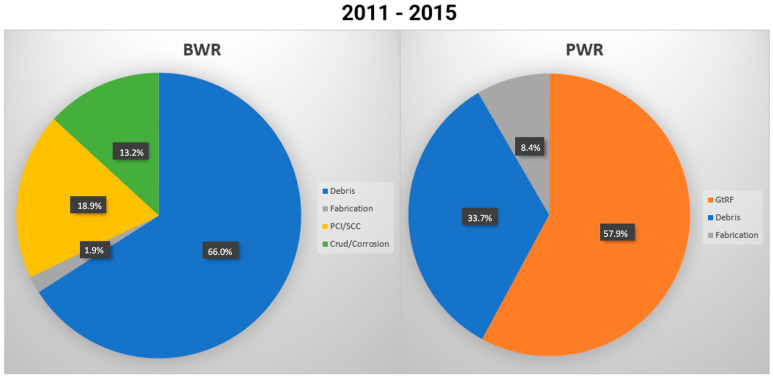
A review of fuel failures in water-cooled reactors (2006–2015), adapted from the EPRI and the zero-fuel failure program, 2010 [4].

Although fuel rod damage has been studied for many years, it remains a significant issue. While the exact causes of leaks are difficult to pinpoint, the most probable cause has been identified with a high degree of certainty. As shown in Figure 1, debris fretting leads to various defects in fuel rods. However, not all causes can be directly detected during standard fuel inspections at nuclear power plants; therefore, more detailed examinations must be conducted in hot cells [1,2].

Debris fretting [3] is primarily caused by coolant flow, which induces flow-induced vibration (FIV). According to a report by Nuclear News in cooperation with the Electric Power Research Institute (EPRI), most fuel rod failures in the U.S. in 2010 were attributed to wear [4]. This is linked to the relatively low wear resistance of cladding materials compared to more robust materials such as stainless steel, a difference that can result in debris like peel-offs, wires, and buckles.

Several studies have shown the significant impact of grid-to-rod fretting, especially in pressurized water reactors [5,6,7]. Experience regarding nuclear power plants [8] has confirmed these findings, although it does not negate the effects of debris-fretting damage. Research in this area is limited, as debris fretting is a highly complex and random phenomenon. However, debris fretting remains an important consideration for new fuel assembly designs.

One potential solution is the installation of an anti-debris filter (ADF) in the bottom nozzles of the fuel assembly. This filter can eliminate objects with a diameter of 1 mm or those exceeding a thickness of 0.3 mm and a length of 10 mm [9]. Efforts to further limit the effects of debris jamming have been made through the introduction of the Foreign Material Exclusion (FME) policy, which is being adopted by nuclear power plants in their internal regulations.

Another approach to reducing the impact of debris is the deposition of thin (micron-scale) wear-resistant coatings on fuel cladding. The thin-film deposition process is becoming increasingly popular and presents a promising solution for grid-to-rod and debris scuffing. Advances in materials technology have made it possible to use new, highly wear-resistant materials as coatings. As the thin-film deposition process becomes more accessible and widespread, it is increasingly being seen as a viable solution for mitigating grid-to-rod and debris fretting [10]. Numerous R&D efforts worldwide are focused on identifying the best available materials, and fuel suppliers and research organizations are actively involved in this process [11,12]. The coating layers of Cr and N compounds can serve as a good example in this regard. CrN coatings are known for their high hardness, which significantly enhances wear resistance [13]. Additionally, these coatings have lower friction coefficients in both dry and lubricated conditions, reducing energy loss and minimizing material degradation over time [14].

One of the key challenges in coating R&D is the qualification of materials and expanding our fundamental understanding of their performance in a reactor’s core. Given that their main advantage is their improved wear resistance, coatings appear to be a promising solution for fretting damage. However, there is a notable lack of recent research on debris fretting, despite it being one of the most common causes of fuel rod failure.

The Research Centre Řež (CVR), in collaboration with UJP Praha, a.s. (UJP), has developed its own methodology for debris-fretting tests, and it has a test facility in which multiple tests can be conducted simultaneously with different coating types. These tests can be performed over short or long durations and in various environments (dry or wet) and conditions (e.g., elevated temperatures, a gaseous atmosphere, etc.). The results from these tests can provide valuable insights for the initial evaluation of applied coating materials. This work is devoted to the qualification testing of new types of coatings based on CrN matrices from the perspective of their wear resistance against debris fretting. The tests were conducted under dry conditions and at room temperature. The qualification of such materials is required before subjecting them to practical conditions in order to eliminate the unstable materials under a cycling mechanical load. The coating materials that undergo local delamination in fretting qualification tests are at risk of experiencing full-scale coating delamination under, e.g., LWR testing conditions (up to 15 MPa and 320 °C). In such cases, the deposition procedures, in addition to the material itself, must be investigated before being tested under practical conditions. The unification of the qualification tests, especially when a very small area of the specimen is cycled and involved in the potential degradation process, is a milestone in the optimization of the advanced cladding material research and development.

## 2. Material, Coating, and Testing Infrastructure

The materials selected for the tests were coatings based on a CrN matrix. These coatings offer notable advancements in mechanical wear performance, particularly in high-stress environments such as those encountered in nuclear applications. The high hardness of CrN significantly enhances wear resistance, with studies demonstrating a marked reduction in wear rates compared to uncoated materials or alternative coatings [13]. CrN coatings also exhibit a reduced coefficient of friction, which is beneficial under both dry and lubricated conditions [14]. This reduction in friction minimizes energy dissipation and mitigates material degradation over time.

The incorporation of a chromium transition layer enhances the adhesion of CrN coatings to metallic substrates, such as zirconium alloys, effectively mitigating the risk of delamination. This interface engineering also reduces residual stress, contributing to greater structural integrity and prolonged durability under dynamic mechanical loading. The dense microstructures of CrN coatings impart resistance to crack propagation, a critical factor in preventing coating failure under cyclic stress conditions [13]. Moreover, CrN demonstrates exceptional thermal stability, retaining its mechanical properties at elevated temperatures [14]. This characteristic renders it particularly suitable for applications in nuclear reactor environments, where thermal and mechanical stresses are significant.

**Figure 2:**
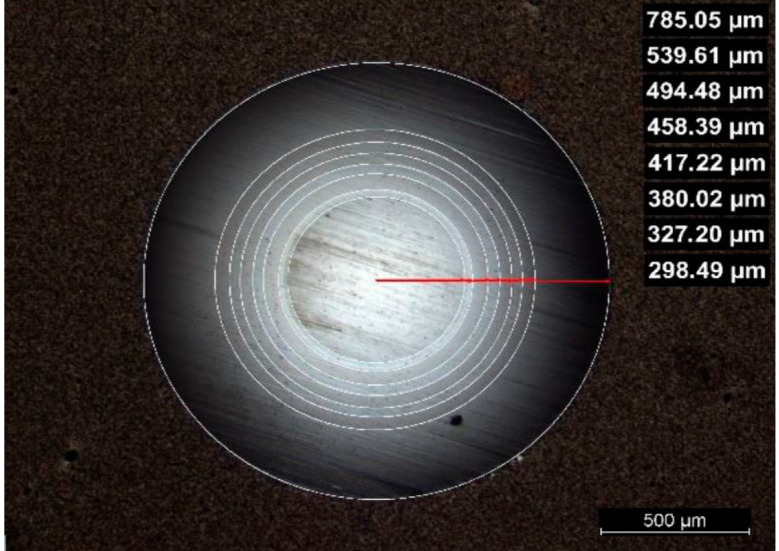
Examination of the polishing of the multi-layer coating on Zr-1%Nb cladding. Red line shows the slope of the measurements’ procedure.

The samples used for the tests were prepared at the Czech Technical University in Prague (CTU) in cooperation with UJP Praha a.s., as described in [15], using the physical vapor deposition (PVD) method. CrN-based coatings were applied to the outer surfaces of Zr-1%Nb tubes using magnetron sputtering in a Hauzer Flexicoat 850 system. The coatings included pure Cr, CrN, and multi-layer Cr/CrN. Deposition occurred at ~250 °C in argon or argon–nitrogen atmospheres, following ultrasonic cleaning and ion etching to enhance adhesion [16]. The coating thicknesses on the cladding material are presented in Table 1, listed from the outermost coating to the substrate. In the same table, the two bottom rows show the multi-layer coating applied to the Zr-1%Nb cladding. This unique coated cladding was further examined using a spherical abrasion test and Scanning Electron Microscopy (SEM) analysis. The results are shown in Figure 2, where the numbers represent a 2D projection of the layer thicknesses obtained during the Calotest.

Depth measurements were conducted by means of a Stylus Profilometer Dektak XT (Bruker), reaching an accuracy on the order of nanometers.

SEM analyses were conducted using a scanning electron microscope (LYRA3 GMU, Tescan, Brno, Czech Republic.).

## 3. Testing Procedure

**Figure 3:**
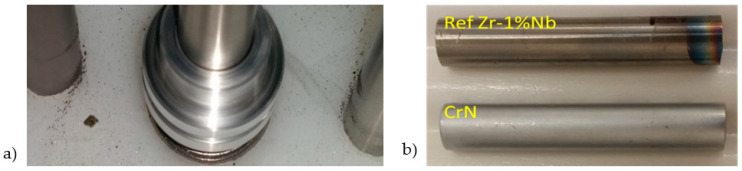
(**a**) Insight into the equipment. Visible pivot with clamped wire and 2 specimens: right—uncoated Zr-1%Nb; left—CrN-coated Zr-1%Nb [17]. (**b**) Full-scale picture of the tested specimens (with a CrN coating layer measuring 16.4 µm).

The CVR developed a testing procedure based on cyclic contact between cladding specimens and a foreign object imitator—a stainless-steel wire mounted on a rotating, shaft-like pivot. The rotating pivot was centrally positioned within the shell-like construction of the test equipment and driven by a motor from the upper end. The barrel at the bottom of the equipment ensured further centralization of the pivot. The specimens were placed at the bottom of the device and fixed in precise positions equidistant from the pivot (Figure 3) in pre-prepared holes in the lower part of the equipment, as described in [17] and [9]. The rotation of the motor was transmitted to the shaft-like pivot, causing the clamped wire to make contact with each specimen.

**Figure 4:**
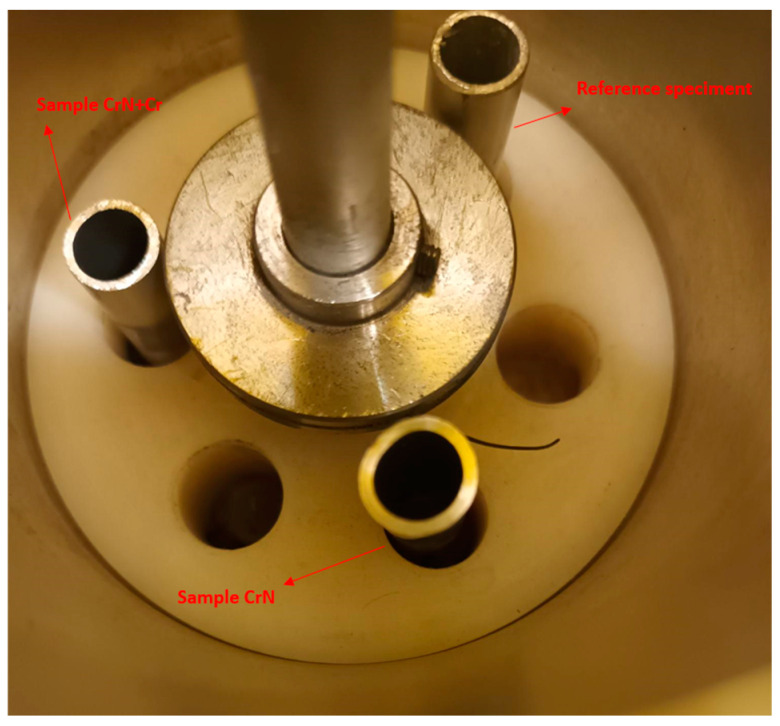
Sample distributions inside the testing equipment.

This design ensured that the free end of the wire made nearly equal contact with each of up to six samples during a single test (as shown in Figure 4). The foreign object imitator—the wire—was bent to ensure smooth contact with the specimen. It was clamped with the mounts on the rotating pivot. The sliding surface of the wire was almost parallel to the surface of the specimen. In this way, the test setup modeled the most conservative scenario—conditions similar to those found inside a reactor core, where a wire (a foreign object that may be left behind during regular maintenance) is trapped by the spacer grid. The free end of the wire was subjected to elevated temperature, pressure, and high coolant flow, causing it to bend in order to minimize friction (i.e., to preserve the lowest resistance).

Given the shape of the wire, the tests conducted at the CVR may have taken longer to break the specimen, but they more accurately replicated real-world conditions. Of course, the FIV ratio of the wire inside the reactor core may have varied depending on the coolant flow, which was highly case-specific. To simplify this problem, the pivot in the equipment rotated at a constant 1000 rpm. The final cycling load placed on the specimens corresponded, in the lowest time regime, to a cycling procedure that was used for the non-nuclear testing of matrix material (with no coating applied) [18]. The wire bent over the pivot was trapped between two clamps, increasing the conservatism and optimizing the test duration. The clamps reduced or eliminated axial vibration of the wire, allowing the cycling impacts to be concentrated on a single area of the specimen [17].

The wire selected for this experiment had a diameter of 0.4 mm, a typical size used in brush heads for maintenance in nuclear power plants [9]. The tests conducted under air conditions served as a pre-characterization of the specimens before autoclave testing, establishing a reference behavior for the specimens, as they could be transferred for further measurements without altering the environmental conditions.

Four cycle time ranges were selected to investigate the effect of the coating application on the fretting resistance of the cladding: 120, 200, 350, and 500 min (and five for the multi-layer tests). These test time ranges were adapted to the capacity of the test equipment and determined through mechanical testing of the system’s auxiliaries. The computer controlled both the impact ratio and the rotation of the pivot. To eliminate the influence of the motor’s own buoyancy, a cycle tracking system was implemented. The 5 V circuit was closed through the reference specimen and the rotating wire, allowing each cycle of direct contact between the wire and the specimen to be counted. This approach provided an almost exact estimate of the number of cycles for each sample.

## 4. Results

All the specimens underwent the cycling described, and the visual examination of the fretting marks is documented in [17]. Three types of tests were conducted, as shown by the reference specimen’s behavior under fretting load. The tests were performed concurrently with the delivery of the samples, as the main objective was to evaluate the fretting resistance of the applied coating.

The depths of the fretting marks were measured using a Stylus Profilometer Dektak XT (Bruker), which features a stylus head with nanometric resolution. Since the applied coatings were in the micron range, the depth measurements of the marks were sufficient. The results fall within the accuracy limits of the system, and all the measurements taken are valid. Measurements were recorded from the surface level of the specimen (0 point), with negative values indicating depth. A potential positive value suggests material deposition in the areas of the fretting mark.

The presented data come from the comparison tests when all the data from one test had to be referenced to the known material (Zr-1%Nb). As each test was thus “case—related”, it was susceptible to small changes in the arrangements; as an example, there could have been a shape change and clamping of the wire. In this way, the outputs had to always be considered as the ratio between the tested material and the reference one.

### 4.1. CrN

**Figure 5:**
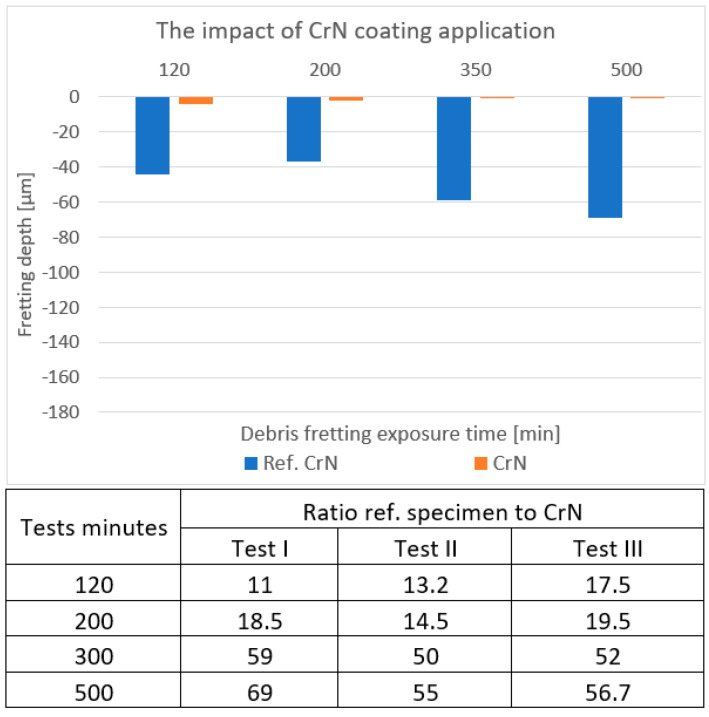
An example of the measured depths on the CrN-coated samples in comparison with the reference samples. The table contains the uncoated/coated depth ratios over three testing cycles.

The CrN-coated specimen and the uncoated Zr-1%Nb specimen were exposed. In Figure 5, one can see the distribution of the obtained marks’ depth in terms of cycling dependency (multiplied by 1000). The blue bars reveal the reference specimens’ depths, while the orange ones refer to those coated with CrN. It is clear that in the case of the coated specimen, no mark exceeded the 5 µm loss against over the 35 µm minimum for the reference specimen. These outputs directly correspond with those from studies indicating wear reductions of up to 37.5 % when optimized deposition methods were used [13].

### 4.2. Cr_x_N _y_

**Figure 6:**
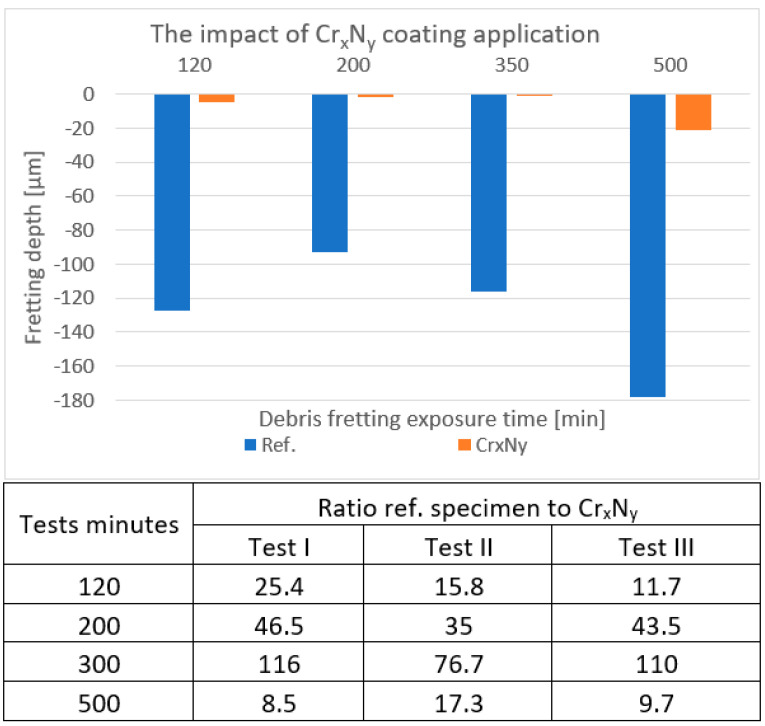
An example of the depths measured on the Cr_x_N_y_-coated samples in comparison to the reference samples. The table contains the uncoated/coated depth ratios over three testing cycles.

Again, the coated specimen was exposed, along with the standard cladding. In Figure 6, the blue lines—corresponding to the reference specimen—exceed 90 µm at a minimum, so the orange lines—corresponding to the coated specimen—stop at around 20 µm. The differences from the CrN coating test derive from the slightly different organization of each particular test and what is visible in regard to the depths of the marks on the uncoated specimen, which is why any coated specimen was always compared with an uncoated one.

### 4.3. CrN+Cr

**Figure 7:**
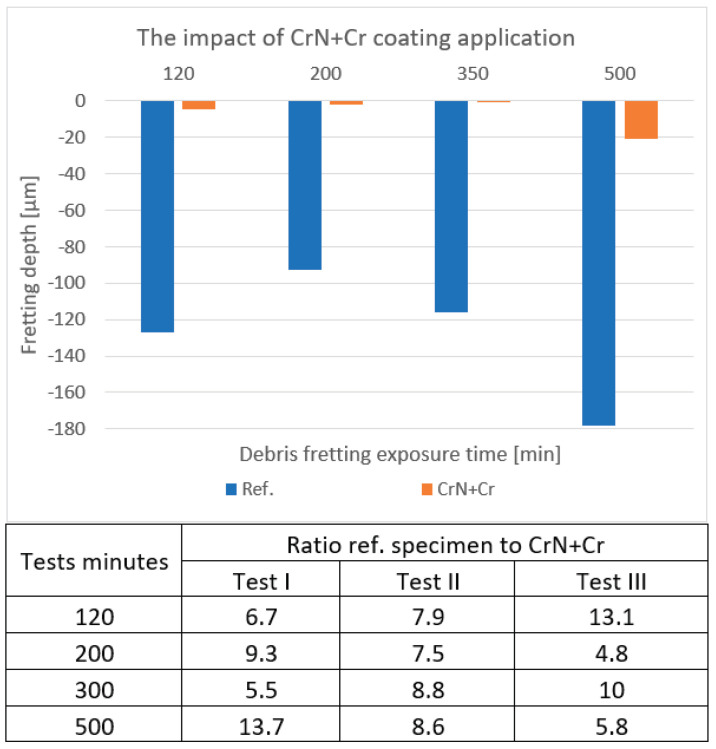
An example of the depths measured on the CrN+Cr-coated samples in comparison to the reference samples. The table contains the uncoated/coated depth ratios over three testing cycles.

A great improvement in fretting resistance is again clearly visible due to the application of the coating on the cladding specimen. The blue marks exceed 80 µm, while the orange ones range up to 20 µm. As one can see, this case is very similar to the Cr_x_N_y_ coating test. The outputs are presented in Figure 7.

### 4.4. Multi-Layer Coating

**Figure 8:**
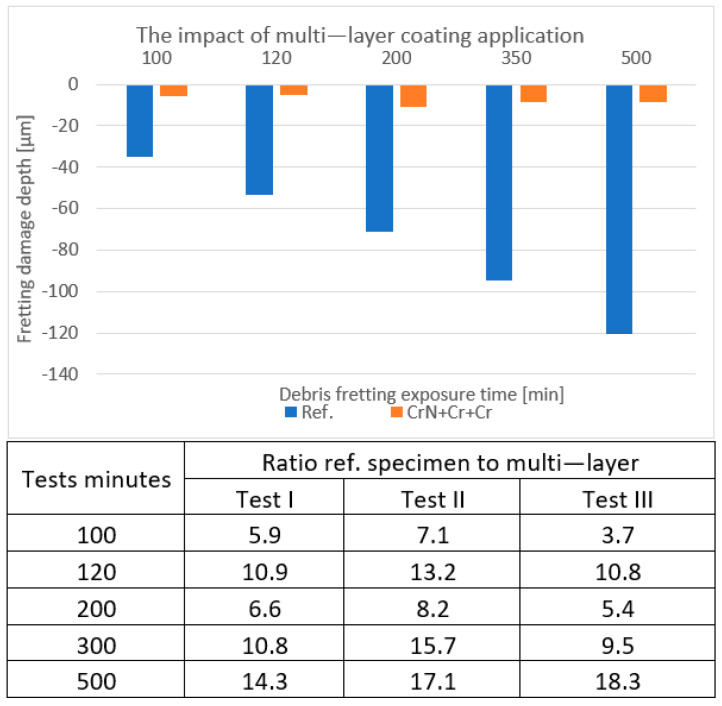
An example of the depths measured on the multi-layer-coated samples in comparison to the reference samples. The table contains the uncoated/coated depth ratios over three testing cycles.

In the case of the multi-layer coating, the outermost layer was one of the thickest, exceeding 10 µm. The marks obtained on this specimen (the orange bars in Figure 8) reached just about 10 µm. In the case of the uncoated specimen, the minimal depth of the fretting marks exceeded 40 µm.

## 5. SEM Analysis Outputs

Two specimens were subjected to SEM analysis: t Zr-1%Nb cladding with CrN and CrN+Cr coatings. The selected samples were subjected to this analysis to verify the coatings’ behavior, with CrN forming the matrix for the outer metallic layer. In both cases, the analysis was conducted after 500,000 cycles.

**Figure 9:**
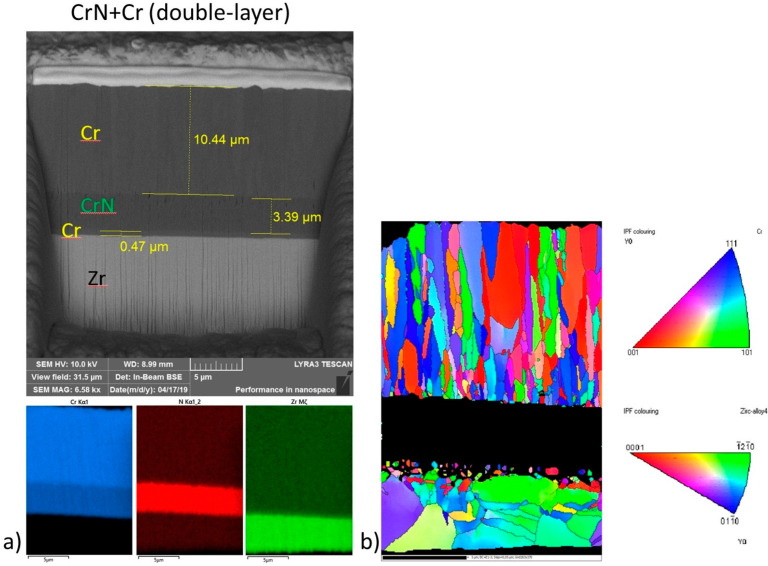
The results of the (**a**) EDS and (**b**) EBSD analyses conducted on the CrN+Cr sample [19].

Between production and the actual tests, the microstructural characterization of the studied samples was performed using Electron Backscatter Diffraction (EBSD) and Energy-Dispersive Spectroscopy (EDS). The results of these analyses for the CrN+Cr bilayer sample are shown in Figure 9. A chemical analysis and an investigation of the elemental composition of the specimens were also conducted during the manufacturing process according to the description in [19].

**Figure 10:**
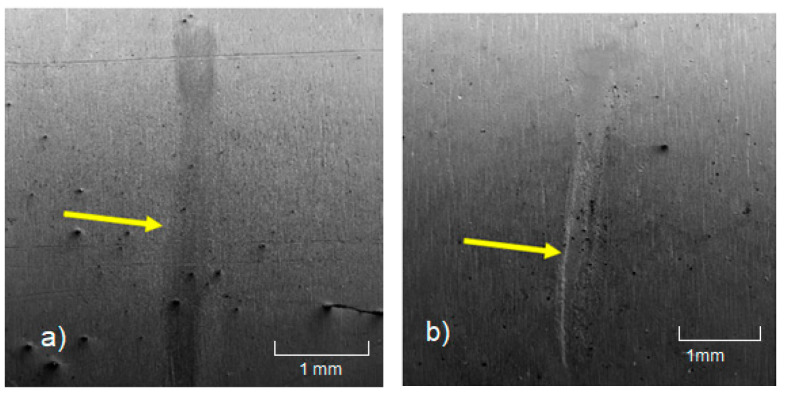
SEM analysis of fretting marks: (**a**) CrN (6 µm); (**b**) CrN+Cr (21 µm).

The SEM+FIB (Focused Ion Beam) analysis visually confirmed the measured maximum depth of the fretting marks. In the latter case, the fretting extended through the Cr layer. An overall image from the SEM analysis is shown in Figure 10. The analysis was conducted at an accelerating voltage of 10 kV in secondary electron (SE) mode.

No deformation or changes to the layers (e.g., delamination) were observed during the SEM analysis. In addition to the SEM analysis, an Energy-Dispersive X-Ray (EDX) spectroscopic analysis was performed, and the results are summarized in Table 2, which shows the elemental maps in the contact area.

## 6. Discussion

All the coating types listed in Table 1 were tested for their debris-fretting resistance. The debris grooves were measured in all cases, and those presented in detail in this paper are consistent with previous results.

It is worth noting that the fretting depth trend exhibited a similar slope coefficient for all the uncoated specimens. This finding indicates the validity of the tests conducted, as the degree of material loss was proportional and comparable across all cases, despite the low wear buoyancy. The highest discrepancy was observed in the case of the CrN specimen. For the multi-layer coating, the trend line showed a smaller discrepancy compared to that for the other specimens. These discrepancies (CrN and multi-layer) were relatively small, and there was no change in the overall trend. The discrepancies arose from the slightly different test arrangements, meaning each test should be compared to the reference specimen.

The endurance of coatings against debris fretting is, in general, a relatively new research area. We expected the coating application to significantly affect the plated specimens, and this expectation was confirmed by the tests. The SEM analysis of the CrN- and CrN+Cr-coated specimens also confirmed the results and showed no changes in the area of the fretting grooves, indicating a successful coating application process. In both cases, the underlying Zr-1%Nb material was unaffected by the fretting.

An interesting observation concerns the second type of coating, CrN+Cr. The debris-fretting test caused the Cr layer (18.6 µm) to be completely worn away in the region of maximum fretting depth. The Cr layer is not as hard as the CrN layer and, as a result, was polished off by the continuous cycling with the stainless-steel wire.

The results from the EDX analysis shown in Table 2 were unexpected. While the presence of elements such as Cr and N within the groove area is not in dispute, as they originated from the coating layer deposited on the Zr-1%Nb specimens, the presence of Fe and Ni particles may be less expected. These particles are directly related to the stainless-steel wire used in the debris-fretting simulation. The wire was prepared from stainless steel, which contains traces of Fe and Ni.

According to the EDX results, the Ni concentration was higher in the case of the CrN+Cr coating, suggesting that this coating exhibited greater toughness, leading to greater material loss from the stainless-steel wire compared to the CrN coating. However, the most interesting finding is the presence of Zr in the groove areas. In all cases, the coating layer did not sustain enough damage to expose the Zr matrix. Furthermore, the distribution of Zr particles was nearly uniform across the entire area of the scuff marks, and there is no evidence of the matrix cladding being released from beneath the coating.

The FIB analysis revealed no traces of Zr deep within the coating layer below the maximum depth of the fretting marks, indicating there was no dissolution of Zr through the coating during the preparation process. Since each test was also conducted parallelly on an uncoated reference sample, the traces of Zr should be associated with the reference specimen. During the tests, small particles of Zr were dislodged from the reference specimen by the rotating wire and distributed over each specimen.

Another factor supporting this conclusion is the slightly increased presence of Zr at the edges of the fretting marks (on both sides). As the tests progressed, the grooves became deeper, and the initial edges were less affected. In the early phase, many Zr particles were removed from the reference specimen, leading to their accumulation in these areas. Following the polishing of the wire a) the amount of Zr residuals decreased (and the friction was lower), and b) the Zr sedimentation in the central area of each groove was constantly affected by the rotating wire; the initial distribution was polished off, and new deposition was limited.

Simulation under dry conditions was selected as the first step in a long-term study concerning fretting damage on nuclear fuel cladding.

## 7. Conclusions

The present work summarizes a wide range of activities in the field of debris-fretting tests performed on standard Zr-1%Nb cladding and coated specimens. The CVR developed a test infrastructure for simulating near-real-life conditions. Since the primary objective was to simulate cycling, a relatively low-force-impact between a foreign object mock-up and several specimens was used for comparison. The test scheme included reference specimens of the matrix cladding material, with the scuff marks obtained on their surface serving as the basis for assessing the influence of the applied coatings. The evaluation was carried out with high precision thanks to constant monitoring of the grooves and their appearance, including via cycle counting, microscopic evaluation of the grooves, and, finally, combined SEM+FIB+EDX analysis.

As expected, the tests confirmed the coating layers had a positive influence on the standard cladding material, particularly in terms of wear resistance. No groove on the coated specimens exceeded a depth of 25 µm, while the minimum depth on the uncoated specimens exceeded 35 µm. In the worst-case scenario, studied at 500,000 cycles, the depth of the coated/uncoated groove was at least 2/72 µm (for CrN). 

In the case of the multi-layer coating, lower fretting resistance of the pure Cr layer was observed, which is consistent with general experience. The wear of the Cr layer, confirmed via both measurements and SEM analysis, did not exceed 10 μm. SEM analysis was also conducted on the CrN sample, and, in both cases, no microstructural changes within the grooves were observed.

The EDX analysis showed that all particles involved in the test procedure were distributed across the grooves, including the deposition of Fe and Nb alongside Cr and N. Furthermore, because the reference specimen was used in each test, the transfer of Zr form the non-coated reference specimen was detected within the fretting marks, although no fracturing was observed.

In the follow-up research, tests are planned to be conducted at elevated temperatures in demineralized water as well as under conditions closely resembling those in LWR reactors (320°C and 15 MPa). In the long term, we plan to transfer the fretting test technology into hot cell facilities for testing active samples.

## Figures and Tables

**Table 1:** The measured thicknesses of the applied coatings.

Coating	Layer 1 (µm)	Layer 2 (µm)	Layer 3 (µm)	Layer 4 (µm)	Layer 5 (µm)	Layer 6 (µm)	Layer 7 (µm)	Layer 8 (µm)	Total (µm)
CrN	16.4								16.4
Cr_x_N_y_	10.7								10.7
CrN+Cr	10.7	18.6							29.3
CrN/Cr+Cr	17.64	0.60	1.25	0.99	1.20	1.15	1.56	10.84	35.23

**Table 2:** Elemental maps of fretting marks on CrN and CrN+Cr (10 mm represents 220 μm).

	CrN	CrN+Cr
Fe K series	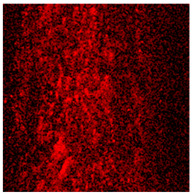	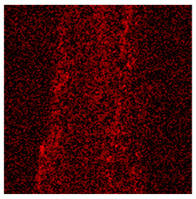
Ni K series	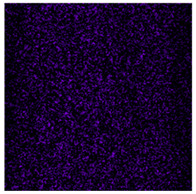	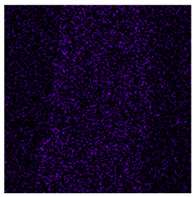
Cr K series	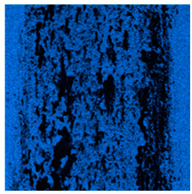	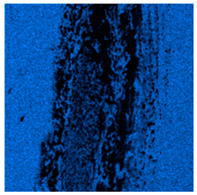
N K series	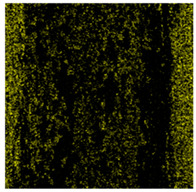	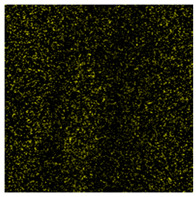
Zr L series	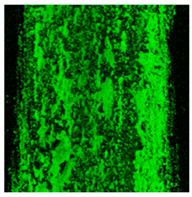	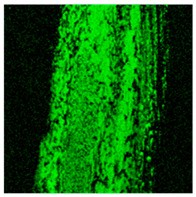

## Data Availability

The data presented in this study are available on request from the corresponding author. The data are not publicly available due to privacy and ongoing further investigation within the project and will be used in further publications.
